# Evaluation of the effects of a generic substitution policy implemented in Chile

**DOI:** 10.1136/bmjgh-2018-000922

**Published:** 2019-03-04

**Authors:** Cristián Mansilla, Jorge Cárdenas, Warren A. Kaplan, Veronika J. Wirtz, Lucy Kuhn-Barrientos, Matías Ortíz de Zárate, Tatiana Tobar, Cristian A. Herrera

**Affiliations:** 1 Ministerio de Salud, Gobierno de Chile, Santiago, Chile; 2 Department of Global Health, Boston University School of Public Health, Boston, Massachusetts, USA; 3 Health Division, OECD, Paris, Île-de-France, France; 4 Department of Public Health, Faculty of Medicine, Pontificia Universidad Catolica de Chile, Santiago, Chile

**Keywords:** health policy, health services research, health systems, health systems evaluation, public health

## Abstract

**Introduction:**

Chile implemented a generic substitution policy in 2014 to improve access to medicines. This study aims to measure if the generic substitution policy had an effect on the sales volume and prices of referent and the branded generic products with demonstrated bioequivalence (BEQ) in the private pharmaceutical market.

**Methods:**

The volume and total private sales of medicines sold at private sector retail outlets between November 2011 and October 2016 were considered in the analysis. We calculated the total number of daily defined doses (DDD) by adding up the number of DDDs of different presentations with the active pharmaceutical ingredient (API). We determined the ratio of the median prices of all BEQ per DDD presentations compared with the median price of the corresponding referent presentations per DDD in 2011 and 2016. Sixteen APIs representing 231 different conventional-release presentations were included in the analysis.

**Results:**

Overall, the volume of sales of the referent products decreased over time after the intervention. However, this reduction was not mirrored by an increase in the corresponding branded generic BEQ volumes overall. In all cases, the median price per DDD of the referent was higher than its BEQ counterpart in 2011 and 2016.

**Conclusion:**

Since referent products are more costly than branded BEQ generic products, reducing their consumption—and increasing the BEQ availability—should improve access to medicines in Chile. However, this must be accompanied by promotion of BEQ products to ensure savings for consumers in the long term. Future research should focus on identifying facilitating and inhibiting factors of generic substitution.

**Key questions**

**What is already known?**

- Policies promoting generic medicines can improve access to medicines. In Chile, a generic substitution policy was implemented to promote the substitution of referent product for generic products.
- No previous study has assessed the effect of the substitution policy in Chile on volume of medicines, using a quasi-experimental method.

**What are the new findings?**

- Overall, the volume of the referent products decreased over time after the intervention. However, this reduction in sales volume was not mirrored by an increase in the corresponding branded generic bioequivalent volumes overall.
- In only 3 of 16 active pharmaceutical ingredients analysed we could find an effect of substitution with branded, bioequivalent, generic product.

**What do the new findings imply?**

- Since referent products are more costly than branded bioequivalent generic products, the reduction in their consumption should improve affordability of medicines in Chile.

## Introduction

Chile has developed several pharmaceutical policies during the last 10 years, aiming at improving the access of medicines. One of the most important policies implemented in 2014 was the generic substitution policy, which was designed to increase market competition by regulating the substitution of generic products for originator medicines (alternatively called ‘referent’).

The impact of policies promoting the uptake of generic medicines have been evaluated in different countries showing their ability to improve access to medicines.^1^ For instance, the introduction of mandatory generic substitution policy in Finland was found to produce a 10.6% decrease in substitutable medicine prices and up to 80% price reduction for some medicines during the first year of implementation.^2^ In the USA, over 10 years from 2002 to 2011, it was estimated that generic medicines saved the healthcare system about US$1 trillion.^3^ Likewise, a cost-minimisation analysis estimated that switching purchasing from 17 innovator brands to the lowest-priced generic equivalents in the private sector of 17 developing countries could result in an average of 60% cost savings.^4^ In this context, generic substitution policies are often promoted as strategies for containing the escalating cost of the medicines.

In Chile, this generic substitution policy regulates the substitution of both branded and unbranded generics. A referent product can be substituted with an unbranded or branded generic product if it contains the same active pharmaceutical ingredient (API), in the same dose and dosage form as the referent product, as well is certified as being bioequivalent (BEQ) to the referent product.

Regulation of the BEQ certification has been introduced through a series of decrees and resolutions in Chile since 1997. These regulations define both the technical aspects related to BEQ and the implementation phases. Each decree released as part of the BEQ regulation includes a list of APIs that must demonstrate the same therapeutic effect as the originator medicine. The decree specifies the due date for demonstrating BEQ for every pharmaceutical product that has an API under this requirement, as well as the product that is being recognised as referent in these BEQ studies (Kaplan *et al*, 2018: Promoting uptake of generic medicines in Chile via bioequivalence certification).

On 14 February 2014, the substitution law started to be implemented, allowing consumers to substitute referent products with BEQ medicines at the point of sale, when both have the same API. This law was the most important pharmaceutical policy that has been implemented in the last decade in Chile. After 3 years of this law, there is uncertainty regarding the effect of this policy. Previous studies have focused on the prices of BEQ products, either before the substitution policy or after its introduction.^5 6^ A difference-in-difference analysis of prices of BEQ versus non-BEQ products between 2009 and 2014 showed mixed results in terms of the prices of the products affected by the policy.^5^ Another cross-sectional study done after the implementation of the substitution policy found that in the private sector, unbranded BEQ products had the lowest price in comparison with branded BEQ; originator products were the most expensive products, on average nine times more expensive than unbranded generic products.^6^ However, no study has assessed the effect of the substitution policy on the volume of medicines, using a quasi-experimental method.

Using interrupted time series analysis (ITS), this study aims to measure whether the generic substitution policy implemented in 2014 in Chile had an effect on the sales volume and price of referent and BEQ generic products in the Chilean private pharmaceutical market. This article is part of the work undertaken under the auspices of the embedded implementation research initiative, which is supported by the Pan American Health Organization and the Alliance for Health Policy and Systems Research, an international partnership hosted by WHO.

## Methods

### Study site

Although Chile has had one of the highest health spending growth among OECD countries between 2010 and 2013, its health per capita spending of US$1877 was less than the average of all OECD countries in 2015.^7^ Also, out-of-pocket (OOP) expenditure, 32.2% in 2015, has decreased in more than 20% between 2005 and 2015,^8^ but it has not yet reached WHO recommendations of 10% to 15%.^9^ Moreover, of the total OOP expenditure, 31% is for medicines.^10^

The Chilean pharmaceutical market is separated into public and private sectors. Public procurement of medicines is mainly driven by an independent public institution (CENABAST), whereas private pharmaceutical market is mainly driven by OOP expenditure (sales of medicines in retail pharmacies).^10^ Since an important part of the total pharmaceutical expenditures is OOP, the private market plays an important role. This study focuses exclusively on the retail private market.

## Data sources

We used two sources of data for this study: (1) retail private market sales database and (2) the market authorisation registry, which is managed by the National Institute of Public Health (ISP).

The private market sales database (database procured by the Ministry of Health of Chile to IQVIA [previously called IMS Health] in Chile) includes information collected between November 2011 and October 2016, on the volume and total private sales (in CLP) of medicines that were sold directly to consumers at the main private pharmaceutical chains of the country (private retail outlets).

This database provides details on every transaction for pharmaceutical products, such as the name of product, API, pharmaceutical presentation, manufacturer, and if it requires being sold with a prescription or not (over the counter).

The national registry of medicines in Chile as per July 2017, which contains information on every market authorisation of medicines in the country, was used to classify each product found in private market sales database as compliant with the BEQ regulation or not.

### Classification of pharmaceutical presentations

Each of the API-containing products presented in the private market were classified into three categories:

- Referent: An API-containing product against which any medicine with the same API is compared with regard to its bioequivalence. Commonly, this is the originator that has conducted clinical studies for its market approval. The specific referent medicine is defined by the ISP using a decree.
- Bioequivalent: It is a branded product that presented the BEQ studies to demonstrate its BEQ before the final BEQ regulation due date (December 2013) set by the decree. It can be also a new branded product that is authorised as a BEQ when it entered the market after December 2013.
- Non-Bioequivalent: Branded pharmaceutical products that did not present BEQ studies or its BEQ study was rejected. These products have never been BEQ during the study period (November 2011–December 2016).

### Selection of APIs

In order to measure the impact of the substitution policy, we selected those 43 APIs that were incorporated into the first decree (Decree no. 500 in 2012). Out of the 43 APIs, we excluded from the analysis 27 APIs, considering the following criteria:

- The daily defined dose (DDD) was not defined (1 API).
- The referent used was a different molecule (1 API).
- The referent defined by the decree was an unbranded generic drug (2 APIs).
- Only modified-release formulations were marketed, which are not affected by BEQ regulations (7 APIs).
- There was an existing patent during the study period (3 APIs), hence there were no generic medicines marketed.
- No generic products were marketed during the study period (12 APIs).
- Product is no longer used, and their sales are negligible (1 API).

We finally included 16 APIs representing 231 different conventional-release presentations in this study. The Online supplementary file 1 shows a list of the total 43 APIs included in this decree with the reasons for exclusions of 27.

10.1136/bmjgh-2018-000922.supp1 Supplementary data

Pharmaceutical presentations that received BEQ certification after December 2013—the date when all products should have received their BEQ approval—were excluded from the analysis because they were not BEQ for the entire time period after the substitution policy intervention date.

We classified each of the 16 APIs according to its therapeutic category, which was extracted from the Anatomic Therapeutic Classification (ATC) at third level, by including the main indication (table 1). The ATC is developed by WHO^11^ and assigns a DDD for each API which we used in our analyses. The DDD is an average daily dose that is defined for each drug in its main indication, which is used to estimate the drug consumption independent of price and dosage form, allowing comparisons between population groups.^12^

**Table 1:** Active pharmaceutical ingredients included in the study by therapeutic group

	Therapeutic category	Active pharmaceutical ingredients
1	Antibiotics	Cefadroxil
2		Doxycycline
3	Antidepressants	Clomipramine
4	Antiepileptics	Clonazepam
5	Antihypertensives	Losartan
6		Verapamil
7	Antithrombotics	Acenocoumarol
8	Antithyroids	Levothyroxine
9	Antiretrovirals	Zidovudine
10	Corticosteroids	Prednisone
11	Hypoglycaemics	Metformin
12	Hypolipidemic agents	Atorvastatin
13	Immunosuppressants	Ciclosporin
14		Mycophenolate
15		Tacrolimus
16	Non-steroidal anti-inflammatory drugs	Ketoprofen

### Data analysis and presentation of results

We used single-group ITS analysis to measure the effect of this policy,^13 14^ without making any seasonality adjustments. We used monthly data and February 2014 as the intervention date. Considering the data from November 2011 until October 2016, we used a total of 60 data points for each time series (27 for the pre-intervention period and 33 for the post-intervention period). We calculated the total number of DDDs (volume) by adding up the number of DDDs of different presentations with the same API.

These analyses were performed in Stata V.14, using the *itsa* command, with the option *newey*. This option estimates coefficients by automatically correcting for autocorrelation and possible heteroscedasticity.^15^

To present the results, we structured the ITS analyses in two levels. First, we present a ‘global’ analysis by summing the consumption volume (sum of DDDs) of all referents and BEQs across all the 16 APIs. Then, we conducted a separate individual ITS analysis for every API referent and BEQ counterpart. Any non-BEQ products sold on the market after the BEQ certification deadline had passed were not considered in our study because the objective of the study was to assess the intended effect of the policy to promote the substitution between referent and BEQ generic products.

We divided our individual analyses into two groups of API-containing products. In group 1 (the ‘two category’ market), we analysed those products whose entire private market in Chile consisted of the referent and its corresponding paired branded generic product. In this group, the corresponding unbranded generic versions had not yet been introduced, or their sales were zero or, at most, 0.05% of the total volume of the market. The seven APIs included in group 1 are verapamil, ciclosporin, tacrolimus, clomipramine, acenocoumarol, zidovudine and mycophenolate mofetil.

In group 2 (the ‘three category’ market), we analysed those paired API-containing products whose market in Chile consisted of the referent and its corresponding branded generic product. However, in contrast to group 1, there also existed at the same time an unbranded generic medicines market with an unknown number of API-containing BEQ products. Thus, this group 2 analysis is only a partial picture of the private pharmaceutical market (since it does not have information on unbranded generics) during our study period. The following nine APIs were included in this group: atorvastatin, cefadroxil, doxycycline, losartan, ketoprofen, levothyroxine, prednisone, metformin and clonazepam.

For every ITS analysis, we report the following coefficients, with the 95% CI and its p value (we considered as statistical significant p values less than 0.05) as presented:

- Level before the substitution policy ($\beta_{0}$).
- Trend before the substitution policy ($\beta_{1}$).
- Level change after the substitution policy ($\beta_{2}$).
- Trend change after the substitution policy ($\beta_{3}$).

In addition to the volume analysis, we compared the ratio of the prices of branded generic BEQ alternative with the referent and analysed if this relationship changed over time. To do this, first we calculated the median price per DDD and its IQR of all referents and BEQ that contain the same API for 2011 and 2016. The prices were obtained from the same private market sales database from which we obtained volume data. We converted the price values to USD using the average exchange rate from CLP to USD in 2011 and 2016.

We then calculated the ratio of the median prices per DDD of the BEQ presentations, compared with the median price of the corresponding referent presentations per DDD, in 2011 and in 2016. This ratio is presented as a percentage of the BEQ median price over the referent price in each year.

### Sensitivity analysis

In addition to the ITS analyses for the trends of each API included in this study, we conducted a sensitivity analysis to explore the effects that this policy could have had on the volume of the APIs that were not included in the BEQ decree.

To do so, we selected APIs that were unaffected by the substitution policy. We chose those APIs that are therapeutically equivalent to the nine APIs in group 2 but which are not included in any decree. In other words, those APIs did not need to demonstrate BEQ testing. The 16 APIs that matched these criteria are listed in the online supplementary file 2. We conducted a time series analysis for those 16 APIs as we did for those 9 APIs included in group 2.

## Results

**Figure 1:**
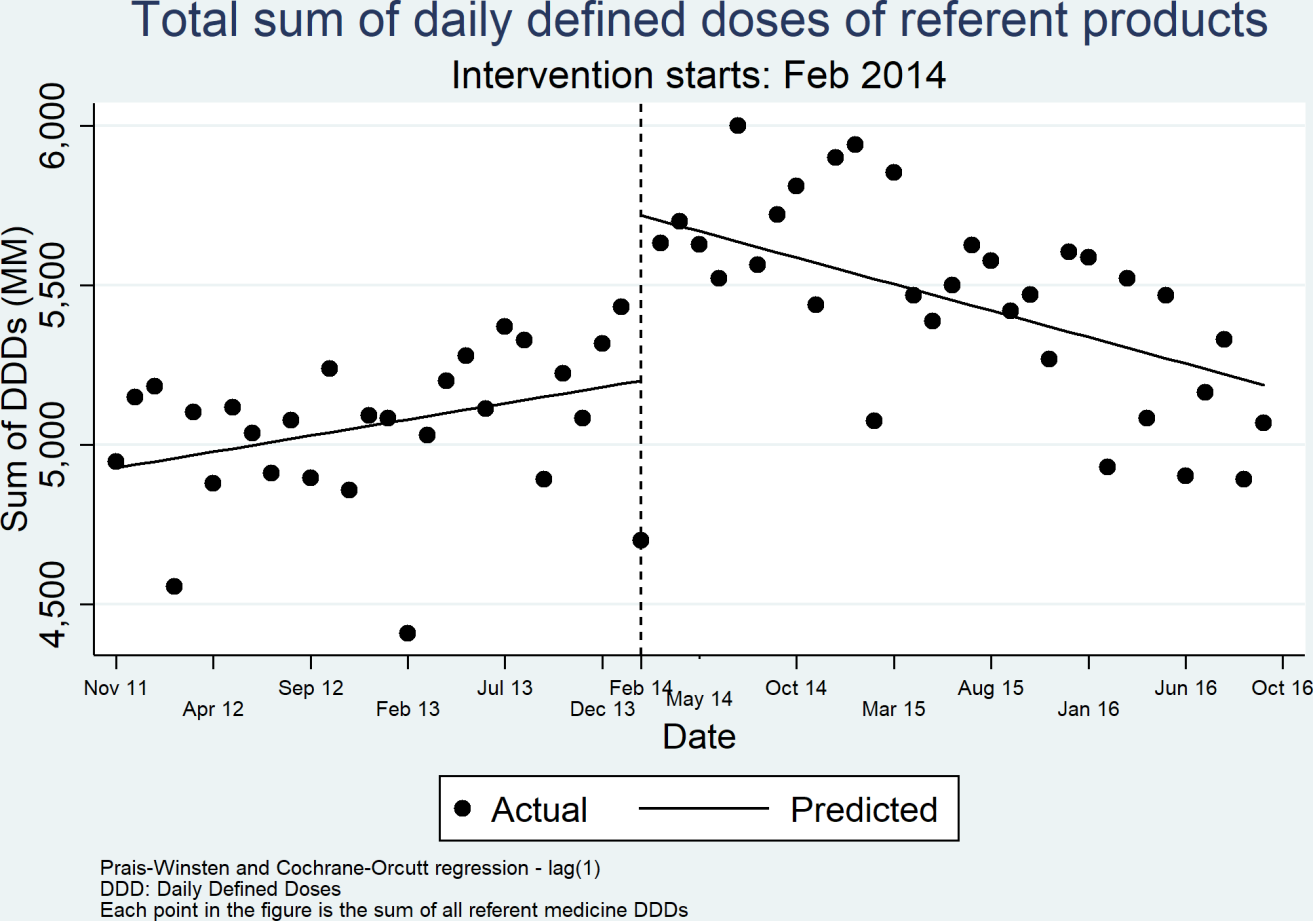
Interrupted time series analysis of the total volume (sum of DDDs) of referent medicines. February 2014 is used as the intervention date.

The 16 APIs that were finally analysed from Decree 500 using ITS represented 231 different conventional-release presentations that were sold in the private market during the study period. Figure 1 shows the ITS ‘global’ analyses of the total consumption volume (sum of DDDs) for referent medicines between 2011 and 2016.

There is a significant increase in the volume of referent medicine DDDs immediately after the intervention followed by a significant change in its slope, showing a reduction of the DDDs of referent after the intervention date (figure 1 and table 2). The model shows an R^2^ of 68.81%.

**Table 2:** Interrupted time series analysis parameters of the total volume for referent and bioequivalent medicines

Active pharmaceutical ingredient	Referent			Bioequivalents		
	Estimate	95% CI	P value	Estimate	95% CI	P value
Total trend						
Level before the intervention ($\beta_{0}$)	4916.8	4786.3 to 5047.3	0.00	2142.8	1767.8 to 2517.7	0.00
Trend before the intervention ($\beta_{1}$)	10.1	1.65 to 18.61	0.02	28.08	0.8 to 55.4	0.04
Level change after the intervention ($\beta_{2}$)	518.5	302.78 to 734.2	**0.00**	−58.3	−722.3 to 605.7	0.86
Trend change after the intervention ($\beta_{3}$)	−26.7	−40.7 to −12.8	**0.00**	−9.7	−48.7 to 29.4	0.62

We are considering a p value less than 0.05 as a statistically significant value. Bold means p value <0.05.

Units used in this analysis are the number of daily defined doses of the active pharmaceutical ingredients included.

In contrast, the total DDDs of BEQ medicines did not show any significant changes, neither immediately after the intervention (level) nor in the slope of their trends (table 2).

### Group 1: ‘two category’ market

Table 3 shows the results of the ITS analyses for every API in this group. In two of seven referent APIs (ciclosporin and acenocoumarol), there was a statistically significant decrease in level of consumption volume of DDDs post-intervention. In the case of BEQ APIs, the change of level post-intervention was significant for ciclosporin and clomipramine, both showing a decrease in their consumption volumes. Although the results are variable, decreases in uptake of BEQ are not correlated with increases in the referent.

**Table 3:** Interrupted time series analysis parameters of the volume of medicines in group 1 (‘two category’ market)

Active pharmaceutical ingredient	Referent			Bioequivalents		
	Estimate	95% CI	P value	Estimate	95% CI	P value
Zidovudine						
Level before the intervention ($\beta_{0}$)	0.60	0.47 to 0.74	0.00	Too few points before the intervention		
Trend before the intervention ($\beta_{1}$)	−0.01	−0.02 to 0	0.22			
Level change after the intervention ($\beta_{2}$)	−0.26	−0.66 to 0.14	0.19			
Trend change after the intervention ($\beta_{3}$)	Product disappeared from the market after the intervention date					
Verapamil						
Level before the intervention ($\beta_{0}$)	0.66	0.62 to 0.71	0	4.94	4.44 to 5.44	0.00
Trend before the intervention ($\beta_{1}$)	−0.01	−0.01 to −0.01	0.00	0.01	−0.03 to 0.05	0.56
Level change after the intervention ($\beta_{2}$)	−0.02	−0.08 to 0.03	0.37	0.67	−0.79 to 2.13	0.36
Trend change after the intervention ($\beta_{3}$)	0.01	0.01 to 0.01	**0.00**	0.01	−0.05 to 0.06	0.82
Ciclosporin						
Level before the intervention ($\beta_{0}$)	4.10	3.78 to 4.43	0.00	0.10	0.06 to 0.14	0.00
Trend before the intervention ($\beta_{1}$)	0.00	−0.02 to 0.02	0.92	0.02	0.02 to 0.02	0.00
Level change after the intervention ($\beta_{2}$)	−0.29	−0.53 to −0.04	**0.02**	−0.15	−0.25 to −0.05	**0.00**
Trend change after the intervention ($\beta_{3}$)	−0.01	−0.03 to 0.01	0.45	0.01	0 to 0.01	0.07
Clomipramine						
Level before the intervention ($\beta_{0}$)	3.47	2.89 to 4.05	0.00	2.84	1.83 to 3.86	0.00
Trend before the intervention ($\beta_{1}$)	−0.03	−0.08 to 0.01	0.14	0.06	0.01 to 0.1	0.02
Level change after the intervention ($\beta_{2}$)	−0.25	−0.93 to 0.44	0.47	−1.50	−2.62 to −0.38	**0.01**
Trend change after the intervention ($\beta_{3}$)	0.03	−0.04 to 0.11	0.39	−0.08	−0.15 to −0.01	**0.03**
Tacrolimus						
Level before the intervention ($\beta_{0}$)	3.43	3.19 to 3.67	0.00	2.63	2.37 to 2.88	0.00
Trend before the intervention ($\beta_{1}$)	0.04	0.03 to 0.05	0.00	0.01	0 to 0.02	0.16
Level change after the intervention ($\beta_{2}$)	−0.09	−0.4 to 0.23	0.58	0.07	−0.21 to 0.35	0.63
Trend change after the intervention ($\beta_{3}$)	−0.02	−0.04 to 0	0.06	0.08	0.05 to 0.1	**0.00**
Acenocoumarol						
Level before the intervention ($\beta_{0}$)	109.28	107.3 to 111.25	0.00	30.82	29.99 to 31.66	0.00
Trend before the intervention ($\beta_{1}$)	−0.73	−0.86 to −0.61	0.00	0.70	0.63 to 0.78	0.00
Level change after the intervention ($\beta_{2}$)	−3.57	−6.08 to −1.07	**0.01**	−0.62	−2.79 to 1.55	0.57
Trend change after the intervention ($\beta_{3}$)	−0.23	−0.37 to −0.09	**0.00**	−0.16	−0.28 to −0.03	**0.01**
Mycophenolate mofetil						
Level before the intervention ($\beta_{0}$)	3.69	3.37 to 4	0.00	3.31	2.88 to 3.73	0.00
Trend before the intervention ($\beta_{1}$)	0.04	0.01 to 0.06	0.00	0.15	0.11 to 0.18	0.00
Level change after the intervention ($\beta_{2}$)	0.28	−0.19 to 0.75	0.24	0.38	−0.35 to 1.12	0.30
Trend change after the intervention ($\beta_{3}$)	−0.02	−0.04 to 0.01	0.20	−0.04	−0.08 to 0	**0.05**

We are considering a p value less than 0.05 as a statistically significant value. Bold means p value <0.05.

Units used in this analysis are the number of daily defined doses of the active pharmaceutical ingredients included.

The trend changes in referent volume over time post-intervention were significant but of small magnitude only for verapamil and acenocoumarol, but the trend post-intervention was different between these two APIs. The intervention for verapamil slowed the negative slope of the referent volume, compared with pre-intervention, and for acenocoumarol, the intervention accelerated the negative pre-intervention volume slope.

In the BEQ presentations, only one API (verapamil) was not affected by the policy. Tacrolimus increased its trend after the intervention and three APIs (clomipramine, acenocoumarol and mycophenolate mofetil) decreased the slope post-intervention.

In the case of zidovudine, the DDD volume of the referent eventually stopped in the private market, whereas the DDD consumption of its BEQ started to increase after the intervention date.

### Group 2: ‘three category’ market

Table 4 shows the results of the ITS analyses for every API in this group. Six from the nine referent APIs in this group (cefadroxil, doxycycline, losartan, ketoprofen, levothyroxine and clonazepam) showed a statistically significant change in level and/or volume trend over time after the intervention.

**Table 4:** Interrupted time series analysis parameters of the volume of medicines in group 2 (‘three category’ market)

Active pharmaceutical ingredient	Referent			Bioequivalents		
	Estimate	95% CI	P value	Estimate	95% CI	P value
Atorvastatin						
Level before the intervention ($\beta_{0}$)	401.61	383.86 to 419.36	0.00	406.58	390.96 to 422.19	0.00
Trend before the intervention ($\beta_{1}$)	−3.40	−4.44 to −2.36	0.00	−2.57	−3.56 to −1.58	0.00
Level change after the intervention ($\beta_{2}$)	−16.41	−40.91 to 8.1	0.19	−8.78	−32.87 to 15.3	0.47
Trend change after the intervention ($\beta_{3}$)	0.21	−1.09 to 1.5	0.75	6.28	4.85 to 7.7	**0.00**
Cefadroxil						
Level before the intervention ($\beta_{0}$)	9.53	9.01 to 10.06	0.00	5.42	3.91 to 6.92	0.00
Trend before the intervention ($\beta_{1}$)	−0.03	−0.07 to 0.01	0.13	−0.10	−0.2 to 0.01	0.07
Level change after the intervention ($\beta_{2}$)	−0.95	−1.78 to −0.12	**0.03**	−0.94	−1.6 to −0.27	**0.01**
Trend change after the intervention ($\beta_{3}$)	−0.03	−0.07 to 0.02	0.20	0.15	−0.01 to 0.31	0.07
Doxycycline						
Level before the intervention ($\beta_{0}$)	3.63	2.76 to 4.49	0.00	10.00	3.45 to 16.56	0.00
Trend before the intervention ($\beta_{1}$)	0.04	−0.03 to 0.12	0.26	−0.10	−0.82 to 0.63	0.79
Level change after the intervention ($\beta_{2}$)	−2.16	−3.62 to −0.7	**0.00**	−3.08	−7.95 to 1.79	0.21
Trend change after the intervention ($\beta_{3}$)	−0.09	−0.17 to 0	**0.04**	0.12	−1.08 to 1.32	0.84
Losartan						
Level before the intervention ($\beta_{0}$)	43.77	34.18 to 53.35	0.00	287.66	71.39 to 503.93	0.01
Trend before the intervention ($\beta_{1}$)	4.29	2.26 to 6.32	0.00	16.73	−2.09 to 35.54	0.08
Level change after the intervention ($\beta_{2}$)	−22.68	−27.15 to −18.22	**0.00**	−116.85	−250.14 to 16.44	0.08
Trend change after the intervention ($\beta_{3}$)	−7.62	−11.22 to −4.02	**0.00**	−22.53	−50.9 to 5.84	0.12
Ketoprofen						
Level before the intervention ($\beta_{0}$)	13.72	9.88 to 17.55	0.00	35.37	15.29 to 55.46	0.00
Trend before the intervention ($\beta_{1}$)	−0.27	−0.47 to −0.07	0.01	0.09	−1.29 to 1.47	0.89
Level change after the intervention ($\beta_{2}$)	−1.25	−3.86 to 1.35	0.34	−24.61	−38.81 to −10.4	**0.00**
Trend change after the intervention ($\beta_{3}$)	0.36	0.11 to 0.61	**0.01**	−0.36	−2.12 to 1.4	0.68
Levothyroxine						
Level before the intervention ($\beta_{0}$)	3577.32	3480.93 to 3673.7	0.00	186.45	120.23 to 252.67	0.00
Trend before the intervention ($\beta_{1}$)	17.35	10.97 to 23.74	0.00	10.85	4.66 to 17.04	0.00
Level change after the intervention ($\beta_{2}$)	495.31	328.38 to 662.25	**0.00**	42.70	−71.59 to 156.98	0.46
Trend change after the intervention ($\beta_{3}$)	−21.08	−32.04 to −10.13	**0.00**	2.70	−9.12 to 14.52	0.65
Prednisone						
Level before the intervention ($\beta_{0}$)	56.70	43.44 to 69.96	0.00	777.26	614.23 to 940.29	0.00
Trend before the intervention ($\beta_{1}$)	−1.72	−2.49 to −0.94	0.00	10.86	−1.29 to 23	0.08
Level change after the intervention ($\beta_{2}$)	−1.38	−5.11 to 2.35	0.46	−138.12	−355.94 to 79.7	0.21
Trend change after the intervention ($\beta_{3}$)	Product disappeared from the market after the intervention date			−0.71	−20.81 to 19.38	0.94
Metformin						
Level before the intervention ($\beta_{0}$)	498.85	488.9 to 508.81	0.00	342.48	313.85 to 371.11	0.00
Trend before the intervention ($\beta_{1}$)	−2.82	−3.51 to −2.13	0.00	−5.79	−9.16 to −2.42	0.00
Level change after the intervention ($\beta_{2}$)	−15.98	−32.33 to 0.38	0.06	−7.07	−22.32 to 8.17	0.36
Trend change after the intervention ($\beta_{3}$)	0.64	−0.41 to 1.69	0.23	6.28	−0.49 to 13.04	0.07
Clonazepam						
Level before the intervention ($\beta_{0}$)	202.84	180.77 to 224.91	0.00	72.84	65.23 to 80.46	0.00
Trend before the intervention ($\beta_{1}$)	−2.30	−3.4 to −1.2	0.00	−0.62	−1.06 to −0.19	0.01
Level change after the intervention ($\beta_{2}$)	0.94	−31.9 to 33.79	0.95	−0.67	−7.38 to 6.04	0.84
Trend change after the intervention ($\beta_{3}$)	1.97	0.62 to 3.31	**0.00**	0.34	−0.12 to 0.79	0.15

We are considering a p value less than 0.05 as a statistically significant value. Bold means p value <0.05.

Units used in this analysis are the number of daily defined doses of the active pharmaceutical ingredients included.

Cefadroxil, doxycycline and losartan showed a reduction in their referent DDD volume level, while levothyroxine increased its referent volume level immediately after the intervention. In terms of the trend, referents of doxycycline, losartan and levothyroxine decreased their slope after the intervention; referents of ketoprofen and clonazepam increased; and prednisone referent was no longer found in the private market after the intervention date, leaving only branded BEQ and unbranded generics of prednisone.

The BEQ branded generic counterparts of two APIs (cefadroxil and ketoprofen) showed a statistically significant decrease in the volume level. Only the branded generic BEQ of atorvastatin had a significant trend change, showing an increase after the intervention. The remaining six branded generic BEQ APIs showed no significant changes (doxycycline, losartan, prednisone, levothyroxine, metformin and clonazepam) post-intervention.

### Sensitivity analysis

Table 5 shows the results of the ITS analyses for the 16 APIs chosen for the sensitivity analysis. These are pharmaceutical products with similar therapeutic effect to the nine APIs included in group 2, but not included in any decree to demonstrate BEQ and, hence, should not be affected by the substitution policy. We could not find any effect of the intervention on the level or the trend of volume of this control group.

**Table 5:** Interrupted time series analysis parameters of the volume of medicines included in the sensitivity analysis (control group of alternative medicines of APIs included in group 2)

	Estimate	95% CI	P value
Control active pharmaceutical ingredients			
Level before the intervention ($\beta_{0}$)	2621.26	2366.45 to 2876.07	0.00
Trend before the intervention ($\beta_{1}$)	38.63	23.12 to 54.14	0.00
Level change after the intervention ($\beta_{2}$)	15.90	−470.41 to 502.22	0.95
Trend change after the intervention ($\beta_{3}$)	−15.34	−39.46 to 8.78	0.21

Units used in this analysis are the number of daily defined doses of the active pharmaceutical ingredients included.

### Price analysis

Table 6 shows the ratio of prices between the branded generic BEQ and the corresponding referent for two points: the ratio of the median prices in 2011 and the ratio of median prices in 2016. The online supplementary file 3 shows the median prices and IQRs for BEQ and referent medicines in 2011 and in 2016 that were used to calculate these ratios.

**Table 6:** Ratio of median prices between the bioequivalents and the referent in 2011 and 2016 shown as percentage (the difference between 2011 and 2016 is also shown)

Active pharmaceutical ingredient (1)	Median price ratio BEQ:referent (2011) (2)	Median price ratio BEQ:referent (2016) (3)	Difference between ratio in 2016 and the ratio in 2011 (4)
Group 1			
Ciclosporin	85.7%	66.1%	−19.6%
Verapamil	46.1%	57.8%	11.7%
Tacrolimus	81.9%	66.4%	−15.5%
Clomipramine	49.5%	45.7%	−3.9%
Acenocoumarol	38.7%	44.5%	5.8%
Mycophenolate mofetil	59.3%	48.7%	−10.6%
Zidovudine	BEQ not sold	Referent not sold	
Group 2			
Atorvastatin	56.1%	38.0%	−18.0%
Cefadroxil	86.4%	87.9%	1.5%
Doxycycline	63.3%	57.9%	−5.3%
Losartan	47.4%	43.4%	−4.0%
Ketoprofen	34.9%	49.6%	14.7%
Levothyroxine	48.0%	75.1%	27.1%
Prednisone	50.4%	Referent not sold	
Metformin	73.0%	76.2%	3.3%
Clonazepam	71.2%	64.9%	−6.3%

BEQ, bioequivalent.

In all cases, the price per DDD of the referent was higher than its BEQ counterpart in 2011 and 2016 (see table 6: ratios <100% in columns (2) and (3)).

In group 1, only two APIs (verapamil and acenocoumarol) showed an increase in the ratio of the price of the BEQ versus the referent between 2011 and 2016, whereas the ratios of the rest of group 1 APIs decreased, meaning that the price of the BEQ decreased further compared with its referent.

In group 2, only ketoprofen, levothyroxine and metformin (cefadroxil had negligible increases) showed increased ratios in 2016, compared with 2011, whereas atorvastatin, doxycycline, losartan and clonazepam decreased their ratios during the same period.

## Discussion

Our study provides important evidence about the effects of the generic substitution policy implemented in Chile on 16 different APIs. Overall, the volume of the referent products decreased over time after the policy intervention. However, this reduction in sales volume of the referent products was not mirrored by an increase in the corresponding branded generic BEQ volumes overall (table 2). The sensitivity analysis shows that, during the time of the study period, there were no significant external changes that could explain these results (table 5). Analysing by API, we did not observe any volume substitution of referent medicines by its branded generic BEQ version except for 3 of the 16 APIs (prednisone, zidovudine and doxycycline) where the referent volume was substituted by the volume of the corresponding BEQ products. This is consistent with the results found in the literature.^16 17^

Significantly, the median BEQ price per DDD was smaller than the referent price per DDD for every API, which means that a substitution of referent for BEQ would have been economically desirable in any case. It is important to note that in general, its price in relation to the originator did not increase. In other words, the increased costs of BEQ testing did not result in higher prices of branded BEQ products in comparison with referent products.

There are numerous factors that could explain the fact that we did not observe a large increase in uptake of BEQ medicines. For example, in Chile the generic substitution is not mandatory for a pharmacy, and needs to be requested by the patient at the point of sale, which might be an important factor for the success of these types of policy.^18 19^ Also, physicians do not have an obligation to prescribe a generic to the patient, which might also have an important impact to the substitution of medicines.^20^ Finally, a communication strategy to promote the use and safety of generic medicines was not conducted at the same time as the substitution policy implementation. This might be an important implementation strategy since prescribers and patients are commonly hesitant regarding the use of generic medicines.^21–24^

One of the main challenges of the policy-making process is to evaluate the policy actions that have been already implemented without proper baseline assessment.^25^ Evaluations of generic substitution policies have been mainly focused on the impact on stakeholder’s views and perceptions.^1 23 24 26 27^ However, there is an evidence gap on the effects of the substitution policy on generic consumption and its effect on medicines prices paid by consumers in Chile.

We recognise several limitations. First, the ‘group 2’ unbranded generics could not be included in our ITS analysis as they could not be disaggregated into BEQ or non-BEQ. Future studies should explore ways to distinguish unbranded generics as BEQ and non-BEQ. Availability of consumption data is critical to evaluate many policies that affect medicines use.

Second, although we explored the generic substitution in each API included in the study, a substitution across different APIs in the same therapeutic category (eg, atorvastatin for simvastatin) is also possible. As a result, an increase or reduction of the volume in one API could be matched with a change in a similar API in the same therapeutic category. However, since the decrees do not necessarily include more than one API of a therapeutic group, we did not analyse APIs by therapeutic group.

Third, only BEQ pharmaceutical presentations that received their BEQ certification before the deadline set by the decree were included. As a result, we cannot explore the effect of this policy on the market entry of new competitors with the same API.

Also, the analyses performed were not adjusted by seasonality. However, only two two APIs had noticeable seasonal trends (prednisone and clomipramine).

Additionally, the sensitivity analysis includes only APIs that are therapeutic alternatives to the products included in group 2. We selected this group since they should have been unaffected by the substitution policy; we are not aware of any significant other policy change (eg, change in clinical guidelines) which could have affected a change in their consumption. For several other APIs that we could have potentially included in the sensitivity analysis, we could not rule out a change in the clinical guidelines.

Finally, there are certain limitations to our private sector medicine data as well. IQVIA (formerly IMS Health) collects data from audits/surveys in the distribution channels of a country, and these channels may change over time and there may be private sector channels in which data are not collected. Clearly, changes in volume and price based on such supply chain audits is not actual patient consumption. Nevertheless, the major strength of these particular data in Chile is that it is indeed capturing most of the private market.^28^

## Conclusions

Overall, there was a reduction in the consumption of referent products over time after the introduction of the generic substitution policy. Since referent products are commonly more costly than branded BEQ generic products (table 6), the reduction in their consumption should potentially improve affordability of medicines in Chile, as long as the availability of BEQ presentations increases. Future research should focus on facilitating and promoting lower cost BEQ products, identifying inhibiting factors of substitution and exploring the impact of the substitution policy in the public procurement of medicines.
