# Supplementary material for: Evaluation of the effects of a generic substitution policy implemented in Chile
Source: BMJ Glob Health. 2019 Mar 4;2(Suppl 3):e000922. doi: 10.1136/bmjgh-2018-000922 (PMC6407566; doi:10.1136/bmjgh-2018-000922)
Supplement: Supplementary data [file bmjgh-2018-000922supp001.pdf]

Supplementary File 1. List of the 43 active pharmaceutical ingredients included in the decree and whether they were included or excluded in the study

Therapeutic category	Molecule	Included or excluded	Reason for exclusion
Anti-arrhythmic	Digoxine	Excluded	The referent defined in the decree were already unbranded generics
Antibiotic	Cefadroxil	Included	N/A
Antibiotic	Ciprofloxacin	Excluded	The referent was not sold during the study period
Antibiotic	Cloxacillin	Excluded	The referent was not sold during the study period
Antibiotic	Doxycycline	Included	N/A
Anticholinergic	Biperiden	Excluded	No longer sold in the Chilean pharmaceutical market
Antidepressant	Clomipramine	Included	N/A
Antiepileptic	Carbamazepine	Excluded	BEQ medicines were not sold during the study period
Antiepileptic	Clonazepam	Included	N/A
Antiepileptic	Phenytoin	Excluded	Did not have sales of any pharmaceutical product with conventional release
Antiepileptic	Valproic acid	Excluded	Did not have sales of any pharmaceutical product with conventional release
Vasodilator	Isosorbide dinitrate	Excluded	The referent defined in the decree was a different molecule (isosorbide mononitrate)
Antihistaminic	Chlorphenamine	Excluded	The referent was not sold during the study period
Antihypertensive	Losartan	Included	N/A
Antihypertensive	Verapamil	Included	N/A
Antineoplastic	Imatinib		DDD is not defined
Antithrombotic	Acenocoumarol	Included	N/A
Antithyroid	Levothyroxine	Included	
antiretroviral	Abacavir	Excluded	BEQ medicines were not sold during the study period
antiretroviral	Atazanavir	Excluded	Existing patent during the study period
antiretroviral	Efavirenz	Excluded	Existing patent during the study period
antiretroviral	Stavudine	Excluded	Did not have sales of any pharmaceutical product with conventional release
antiretroviral	Fosamprenavir	Excluded	Did not have sales of any pharmaceutical product with conventional release

antiretroviral	Indinavir	Excluded	Did not have sales of any pharmaceutical product with conventional release
antiretroviral	Lamivudine	Excluded	BEQ medicines were not sold during the study period
antiretroviral	Nevirapine	Excluded	BEQ medicines were not sold during the study period
antiretroviral	Ritonavir	Excluded	BEQ medicines were not sold during the study period
antiretroviral	Saquinavir	Excluded	Did not have sales of any pharmaceutical product with conventional release
antiretroviral	Tenofovir disoproxil	Excluded	Existing patent during the study period
antiretroviral	Zidovudine	Included	N/A
Corticosteroid	Prednisone	Included	N/A
Diuretic	Furosemide	Excluded	The referent was not sold during the study period
Gonadotropin	Clomifene	Excluded	BEQ medicines were not sold during the study period
Hypoglycemic	Glibenclamide	Excluded	The referent was not sold during the study period
Hypoglycemic	Metformin	Included	N/A
Hypolipidemic	Atorvastatin	Included	N/A
Immunosuppressant	Cyclosporine	Included	N/A
Immunosuppressant	Methotrexate	Excluded	The referent defined in the decree were already unbranded generics
Immunosuppressant	Mycophenolate mofetil	Included	N/A
Immunosuppressant	Tacrolimus	Included	N/A
NSAID	Diclofenac	Excluded	Did not have sales of any pharmaceutical product with conventional release
NSAID	Ketoprofen	Included	N/A
Propulsive	Metoclopramide	Excluded	The referent was not sold during the study period

Legend: N/A= not applicable

From the original list of 43 APIs, we excluded one active pharmaceutical ingredient (API) whose Daily Defined Dose (DDD) is not specified in the WHO registry, namely imatinib, which probably does not have a DDD due to the different indications depending on the type of cancer. We further excluded 3 APIs where the referent was defined as a different molecule than the API included in the decree (e.g isosorbide dinitrate versus isosorbide mononitrate), or the referents defined in the decree were

already unbranded generics (digoxin and methotrexate). Later, we excluded 7 APIs that did not have sales of any pharmaceutical product with conventional-release (phenytoin, diclofenac, fosamprenavir, indinavir, saquinavir, stavudine and valproic acid: found only in modified-release). Also, 3 APIs under an existing patent during the study period were excluded: atazanavir (currently under patent), tenofovir (patent expired during 2017), and efavirenz (patent expired during 2014). We further excluded 6 APIs in which the referent was not sold during the study period (metoclopramide, glibenclamide, chlorphenamine, cloxacillin, furosemide and ciprofloxacin), and 6 APIs that, although the referent was sold, we could not find sales of bioequivalent medicines during the study period (abacavir, lamivudine, nevirapine, ritonavir, clomiphene, and carbamazepine). Finally, we excluded 1 API whose sales level was very small and, in fact, is no longer sold in the Chilean market (biperiden).

Supplementary File 2. Active Pharmaceutical Ingredients that were used for the sensitivity analysis.

API included in the study	APIs considered as alternatives
**Atorvastatin**	Ezetimibe<br>Pitavastatin<br>Pravastatin<br>Rosuvastatin
**Cefadroxil**	Amoxicillin<br>Ampicillin<br>Flucloxacillin
**Doxycycline**	None
**Clonazepam**	Clobazam<br>Clorazepate
**Ketoprofen**	Dexibuprofen<br>Flurbiprofen<br>Ketorolac
**Levothyroxine**	None
**Losartan**	Lisinopril
**Metformin**	None
**Prednisone**	Dexamethasone<br>Hydrocortisone<br>Methylprednisolone

Supplementary File 3. Median Prices and Interquartile ranges of bioequivalent and referent medicines in 2011 and 2016.

ACTIVE PHARMACEUTICAL INGREDIENT	Median price of BEQ in 2011 in USD (IQR)	Median price of BEQ in 2016 in USD (IQR)	Median price of referent in 2011 in USD (IQR)	Median price of referent in 2018 in USD (IQR)
**GROUP 1**				
Ciclosporin	15.67 (15.64 , 15.69)	18.28 (18.25 , 18.31)	8.98 (8.63 , 9.28)	13.65 (13.57 , 13.69)
Verapamil	2.57 (2.54 , 2.59)	5.57 (5.54 , 5.6)	2.34 (2.22 , 2.45)	4.06 (3.93 , 4.07)
Tacrolimus	26.702 (26.701 , 26.704)	32.59 (32.56 , 32.62)	13.91 (12.06 , 15.8)	21.65 (21.35 , 22)
Clomipramine	3.98 (3.94 , 4.03)	8.04 (8 , 8.09)	2.98 (2.96 , 3.09)	6.53 (6.09 , 6.75)
Acenocoumarol	1.06 (0.74 , 1.38)	2.73 (2.72 , 2.74)	0.93 (0.44 , 1.14)	2.09 (2.07 , 2.1)
Mycophenolate mofetil	12.16 (8.4 , 15.93)	20.64 (20.6 , 20.69)	6.18 (5.43 , 8.76)	12.68 (12.57 , 12.88)
Zidovudine	Not sold	9.27 (9.25 , 9.29)	3.7 (3.58 , 3.88)	Not sold
**GROUP 2**				
Atorvastatin	1.07 (0.78 , 1.26)	1.91 (1.9 , 1.92)	0.46 (0.30 , 0.88)	1.23 (1.22 , 1.24)
Cefadroxil	8.91 (8.87 , 8.96)	10.31 (10.26 , 10.37)	6.72 (6.68 , 6.77)	7.64 (7.57 , 7.82)
Doxycycline	2 (1.99 , 2.004)	3.16 (3.14 , 3.18)	1.39 (1.38 , 1.4)	2.41 (2.397 , 2.421)
Losartan	0.552 (0.391 , 0.689)	1.165 (1.152 , 1.178)	0.358 (0.114 , 0.625)	0.898 (0.896 , 0.92)
Ketoprofen	0.98 (0.71 , 1.26)	2.89 (2.89 , 2.89)	0.8 (0.67 , 0.89)	1.6 (1.59 , 1.6)
Levothyroxine	0.259 (0.258 , 0.26)	0.54 (0.39 , 0.7)	0.24 (0.23 , 0.24)	0.32 (0.31 , 0.33)
Prednisone	0.288 (0.285 , 0.29)	0.57 (0.56 , 0.58)	0.19 (0.18 , 0.2)	Not sold
Metformin	0.8 (0.46 , 0.89)	1.1 (1.04 , 1.16)	0.73 (0.61 , 0.82)	1 (0.95 , 1.04)
Clonazepam	2.44 (1.72 , 2.54)	3.42 (3.4 , 3.45)	1.71 (1.23 , 1.74)	2.64 (2.62 , 2.64)
